# Mining of miRNAs from EST data in Dendrobium nobile

**DOI:** 10.6026/97320630016245

**Published:** 2020-03-31

**Authors:** Debasish B Krishnatreya, Pooja Moni Baruah, Bhaskar Dowarah, Kuntala Sarma Bordoloi, Heena Agarwal, Niraj Agarwala

**Affiliations:** 1 Department of Botany, Gauhati University, Guwahati, Assam, India - 7810014

**Keywords:** Expressed Sequence Tags, miRNA, in silico, Dendrobium nobile

## Abstract

Dendrobium nobile is an orchid species highly popular for its therapeutic properties and is often used as a medicinal herb. Documenting miRNA-target associations in D. nobile is an important step to facilitate functional genomics studies in this species. Therefore, it is of interest to identify miRNA sequences from EST data available in public databases using known techniques and tools. We report 14 potential miRNAs from three ESTs of D. nobile. They belong to 3 miRNA families (miR390, miR528 and miR414) linking to transcription factor regulation, signal transduction, DNA and protein binding, and various cellular processes covering 34 different metabolic networks in KEGG. These results help in the understanding of miRNA-mRNAs functional networks in Dendrobium nobile.

## Background

Dendrobium nobile is ornamentally and medicinally one of the most important species of flowering plants. It belongs to the Orchidaceae family, which is one of the largest families of the angiosperms and has been used as a first-rate herb in India and China since ancient times [1]. The pattern of flowering of the violet coloured flowers of D. nobile make them more fascinating and attractive [2]. The presence of various active compounds likes Dendrobine, Moscatilin, Gigantol, Nobiline and Dendrophenol in the stems and leaves of D. nobile are known to be responsible for the greatly increased medicinal property of this plant [3, 4]. These compounds are known to have strong anti-mutagenic properties and are anti-carcinogenic against lung carcinoma, ovary adenocarcinoma and promyelocytic leukemia [5]. Moreover genetic diversity studies indicate that D. nobile from Northeast India has a comparatively higher rate of genetic diversity [6,7]. The orchid, being prized for its immense commercial importance, is often subjected to unrestrained anthropogenic pressures, thereby threatening its natural population [8]. In addition to its health benefits and economic value, D. nobile is also a wonderful source of experimental material to expound gene expression and regulation because of its versatile characteristics; the availability of decent numbers of expressed sequence tags of this species also augmented this study.

MicroRNAs are a class of endogenous small, non-coding, single stranded RNAs that act as post-transcriptional regulators in eukaryotic organisms [9]. Each miRNA is capable of regulating the expression of many genes - either by translational repression or mRNA cleavage- allowing them to simultaneously regulate multiple cellular signalling and biosynthetic pathways [10]. Plant microRNAs play important roles in plant growth and development including leaf morphology and polarity, organ development, cell differentiation and proliferation, programmed cell death, signal transduction, stress responses, hormone signalling, floral organ identity and maturity, phase transition and reproduction [11-13]. For miRNAs to be reliably distinguishable from other RNAs, Ambros et al. (2003) developed a set of criteria for miRNA identification and annotation and their guidelines for experimental verification [14]. However, those criteria for miRNA annotation have been revised by Axtell and Meyers (2018), which has been followed in this study [15]. The first miRNA to be discovered was lin-4, predicted to be of 22 nucleotides in length and found in the larval form of Caenorhabditis elegans [16]. It is responsible for regulation of the pathway that triggers the transitions of first larval stage cell division to the second [17]. In plants, RNA polymerase II is responsible to transcribe majority of primary miRNA transcripts (pri-miRNAs) from miRNA genes. Processing of pri-miRNAs to precursor miRNAs and then further to mature miRNA-miRNA* duplex is brought about by the DCL1 (Dicer-like 1) enzyme [18]. The duplex is methylated by HUA ENHANCER 1 (HEN1) and transported to the cytoplasm by HASTY, after which the guide miRNA strand is then incorporated into ARGONAUTE (AGO) protein [19]. Once a suit-able pairing event between a miRNA and target mRNA occurs, the RISC (RNA-induced silencing complex) then triggers almost complete inhibition of protein expression by either cleavage of mRNA targets or by inhibiting protein translation [20]. The repressional activity of miRNA is mainly based on the property of regulation of gene expression at the post-transcriptional level either by cleavage mediated mRNA degradation or inhibition of translation [21]. Discovery of genetic modulators in various plants has helped to comprehend their specific regulatory modules involved in complex biological processes. Understanding the biological functions of miRNAs, identification of miRNAs and their target genes is an important step in interpreting the roles of miRNAs in regulation of specific characters. Documentation of miRNAs and their targets have been very effective in a number of plants such as Arabidopsis, rice, maize, wheat, soybean, cotton and tea [22, 23].

## Methods:

### Reference set of miRNAs and Sequence data:

A total of 38,589 previously identified mature micro-RNAs from different plants were retrieved from the miRBase database (http://www.mirbase.org/) (release 22.1). These sequences were defined as the query sequence set and used for identifying miRNAs in D. nobile Expressed Sequence Tags (ESTs). Publicly available 15,383 ESTs of the species were downloaded from National Centre for Biotechnology Information (NCBI)(https://www.ncbi.nlm.nih.gov/). Local database for BLAST was constructed for D. nobile ESTs by using the locally installed NCBI-Blast+ application (ftp://ftp.ncbi.nlm.nih.gov /blast/ executables/blast+/). Non-redundant protein sequences were used from the NR protein database of NCBI (ftp://ftp.ncbi.nlm.nih.gov/blast/db/).

### Identification of putative miRNAs:

**Figure 1:**
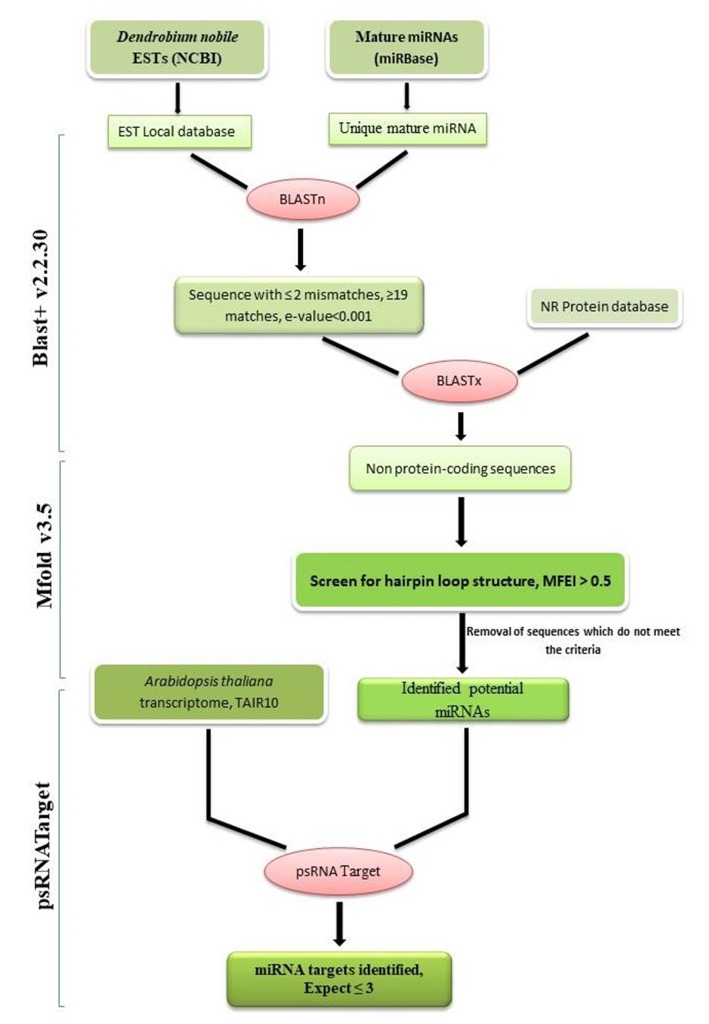
Computational pipeline for identification of putative miRNAs of D. nobile and their target genes

Sequence and structural homologies are used for computer based predictions of miRNAs. Computational strategies provide less time consuming, valuable and efficient means for prediction and identification of miRNA genes and their targets (Figure 1). NCBI-BLAST+ program was used to screen the ESTs against the reference miRNAs obtained from miRBase by searching for homologous hits [24]. A maximum of two mismatches,threshold e-value of <0.001 and word-size value of 7 was set for the blast+ analysis. After removing redundancy the ESTs with matched hits were subjected to Blastx analysis with NR protein database, and the non-protein coding sequences were retained for further analysis of RNA secondary structure using Zuker folding algorithm by Mfoldv3.5 (http://unafold.rna.albany.edu/ ?q=mfold/ RNA-Folding-Form) [25]. The following parameters were used in defining the sequences as miRNA homologs: (1) The sequence should fold into an appropriate stem-loop secondary structure. (2) The miRNA should be present in one arm of the hairpin structure. (3) The mature miRNA and its complementary miRNA* sequence should not have more than 5 mismatches. (4) The value of Minimal Folding free Energy Index (MFEI) of precursor miRNA structures should not be less than 0.5 and should have a high Minimal Folding free Energy (MFE) value. MFE is the negative equivalent of the ΔG value [26]. The MFEI value has been calculated by using the following formula proposed by Zhang et al. [27].

 AMFE = (MFE x 100)/Length of precursor 

 MFEI = AMFE / (GC) % 

 MFEI = [(MFE/length of the RNA sequence) x 100]/(GC)%

### Prediction of putative target genes:

A plant small RNA Target Analysis Server viz. psRNATarget was used for predicting the targets of the newly identified miRNA by using Schema V2 (2017 Release) with the maximum expectation value threshold as 3 and rest of the values set as default [28]. A maximum of two mismatches were allowed in the complementary region of target genes with the miRNAs, whereas mismatch inhibition was maintained at 10th and 11th nucleotide position along the aligned region. Target genes were identified against Arabidopsis thaliana transcript, TAIR V10 as genome or transcriptome sequences of Dendrobiumnobile are not available in public domain.

### Gene Ontology, KEGG pathway and Phylogenetic analysis:

Annotations of the target genes were carried out using a Blastx analysis with an e-value of 10 3 against the NCBI non-redundant protein database. Blast2go version 5.2 (https://www.blast2go.com/blast2go-pro/) was used for the gene ontology and KEGG (Kyoto Encyclopaedia of Genes and Genomes) pathway analysis of the annotated target genes in order to assess the phenotypic traits which may get affected by expression of the identified miRNAs of D. nobile [29]. The phylogenetic trees were constructed using MEGA7 – a Windows OS based software. The precursor sequences of family members of the identified miRNAs, belonging to other plant species were downloaded from miRBase and collated with the D.nobile miRNA precursors. Multiple sequence alignment was carried out using MUSCLE algorithm and phylogenetic trees developed using the Maximum likelihood approach.

## Results:

### miRNA identification and characterization:

From a total of 15,383 published ESTs of D. nobile, 306 of them showed homology with previously deposited miRNAs in miRBase 22.1. Following the criteria given by Axtell and Meyers (2018) for plant miRNA annotation, these were further filtered to retain only the miRNAs ≥ 19 nucleotides in length [15]. As a result only 249 miRNAs were taken from which further removal of redundancies in miRNAs and ESTs yielded 247 potential miRNA sequences. Blastx analysis of these ESTs against the NCBI non-redundant database resulted in identification of 89 sequences as non-coding sequences.

### miRNA secondary structure:

**Figure 2:**
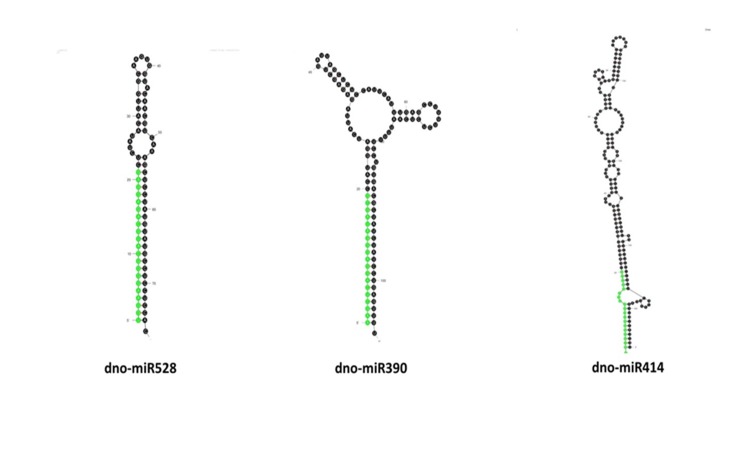
Secondary hairpin structure of precursor sequences of three identified miRNA families.

The potential miRNAs were subjected to structural validation analysis in Mfold v3.5 for prediction of miRNA secondary structure. The miRNAs which showed valid stem-loop hairpin precursor, presence of complementary miRNA* sequence in the precursor with less than 6 mismatches, and an MFEI value greater than 0.5 were considered for further analysis of their target genes. Fourteen such conserved miRNAs were identified belonging to three miRNA families (Figure 2). miR528 is represented by two members, miR390 represented by 11 members and miR414 by one member. The ΔG values ranged from -47.8 to -35.5 kcal/mol. It is often considered that, lower the value of ΔG, higher is the thermodynamic stability of the miRNA precursor [30]. A lower value of ΔG corresponds to a higher MFEI value as MFE is equivalent to (-ΔG). miRNA characterization indicates that the precursor length of miRNAs varied between 79-169 bases and the mature miRNA length ranged from 19 to 21 nucleotides (Table 1).

### Target gene prediction and annotation:

It has been demonstrated in several studies that most plant miRNAs bind to their target mRNA sequences with perfect or near-perfect sequence complementarity [31, 32]. This provides an effective approach for discovering probable miRNA targets by comparing and aligning miRNAs with mRNA sequences. In order to identify genes plausibly recognised by the potential miRNAs, psRNA Target - a web-based server was used for searching target genes against A. thaliana transcriptome acquired from TAIR10. A total of 138 genes were identified as target genes of 14 identified miRNAs, where 4 genes having unknown functions were discarded. Out of the 134 retained targets, only 3 genes exhibit translational repression by corresponding miRNAs whereas all the rest of the genes show cleavage mode of regulation (Table 2).

### GO and KEGG pathway analysis:

**Figure 3:**
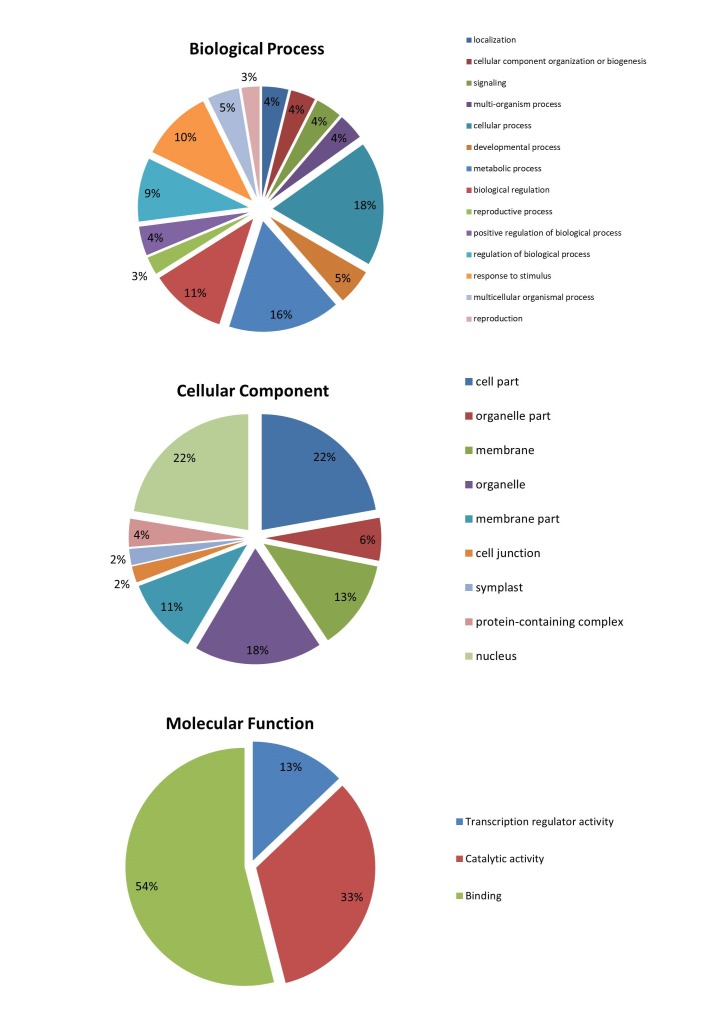
GO reports of the identified target genes showing percentage of sequences representing each class in three different categories viz. Biological processes, Cellular component and Molecular Function

**Figure 4:**
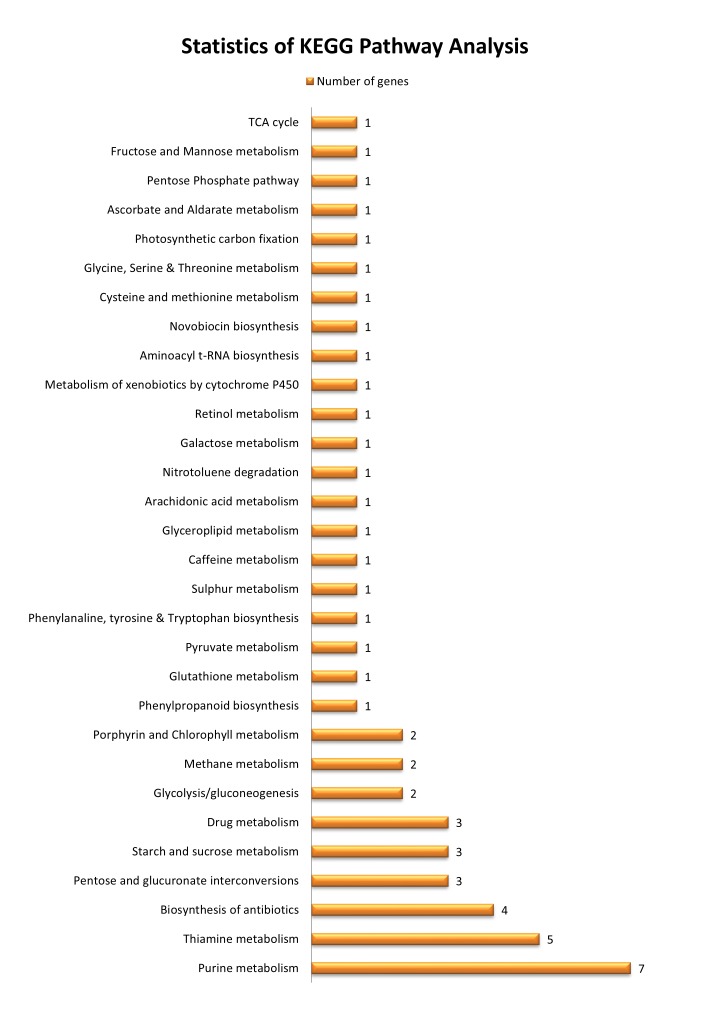
KEGG pathway analysis reports of the target genes showing the number of genes belonging to each pathway.

To further understand the regulatory functions of miRNAs, the target genes were subjected to Gene Ontology (level 2) and KEGG pathway enrichment analysis, using Blast2Go v5.2. The results suggested that D.nobile miRNAs were involved in regulation of 14 broadly defined biological processes and 3 basic molecular functions. The target genes were also found to be part of 9 different types of cellular components (Figure 3). Pathway enrichment analysis of target genes based on KEGG database demonstrated the participation of identified miRNAs in 34 different metabolism networks (Figure 4). These networks are involved in various important pathways such as purine metabolism, antibiotic synthesis, caffeine metabolism, pentose phosphate pathway and TCA cycle.

### Phylogenetic Analysis:

**Figure 5:**
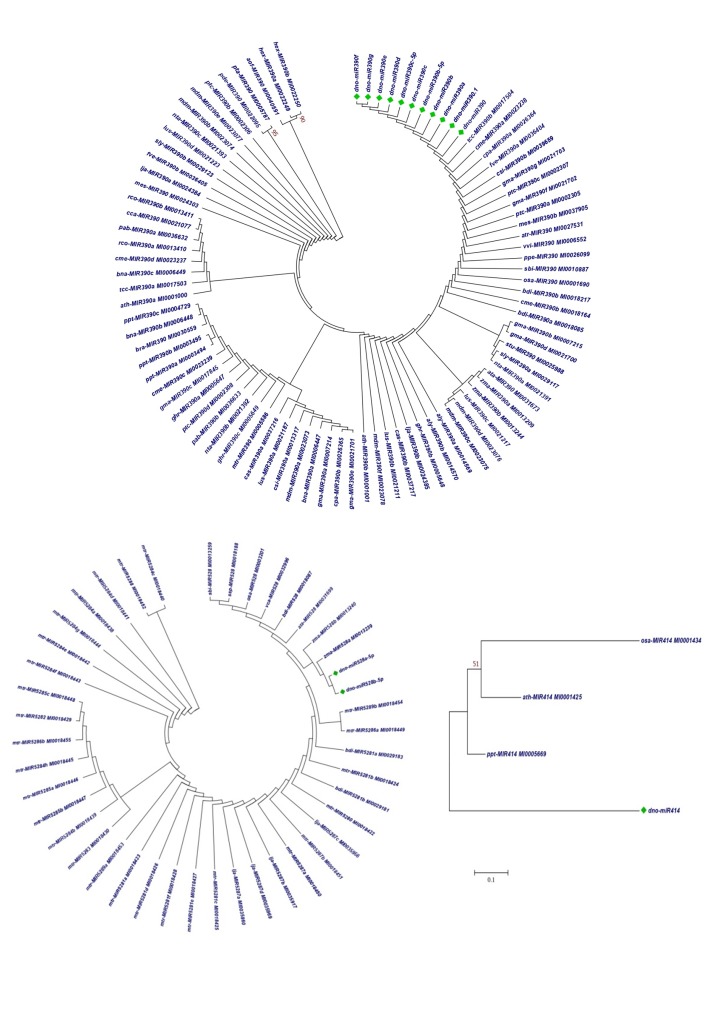
Neighbour joining Phylogenetic trees constructed using stem-loop precursor sequences for three different groups of miRNAs i.e. miR390, miR414 and miR528a and b. Entries marked with green dots have been identified from D. nobile ESTs.

Phylogenetic analysis was carried out to understand the relationship between the identified miRNAs in D. nobile with the other plant species available in miRNA database for same family identification (Figure 5). No miRNAs have been reported for D. nobile in miRBase. Maximum likelihood method was used for carrying out three different phylogenetic analyses for three identified miRNA families and their representative members. miR390 is a conserved miRNA family and its members have reported in many important species including Arabidopsis, Brassica and rice, whereas miR528a and miR528b have been reported only in Zea mays. miR414 have been reported only in three species in miRBase viz. A. thaliana, Oryza sativa and Physcomitrella patens.

## Discussion:

Identification and annotation of genetic modulators help in deciphering the critical roles played by such components in regulation of specific biological processes and their associated cellular properties. miRNA's are considered as one such group of regulatory molecules which inhibit gene expression by cleavage mediated target mRNA degradation or translational repression. Before this study, no comprehensive work was done on identification of putative miRNAs from Expressed Sequence tags of D. nobile. In this research we considered all the important criteria such as the MFEI values, mismatch inhibition and sequence length which have been used for miRNA identification in other angiospermic species. The MFEI values of the 14 identified miRNAs in our work were mostly in the range of 0.5 to 1.0, among which 13 of them have MFEI values even greater than 0.9. As compared to the miRNAs identified in some other plants from EST sequences [33-36],this is a comparatively higher range of MFEI values, and a higher value of MFEI indicates greater thermodynamic stability of the secondary structure of the miRNAs, and hence lesser chance of encountering false positives. The G+C% of most of the miRNAs was found to be in the range of 41-47%, however only the members of miR528 family presented a G+C% value greater than 50. Among the predicted targets 13% genes are sequence specific transcription factors, 33% genes with various catalytic functions and 54% genes act as sequence specific DNA-binding, metal ion binding or protein binding factors. In the gene ontology analysis, the two main categories represented among the biological processes are cellular processes and metabolic processes (18% and 16% genes respectively). 22% of the target proteins have been found to be part of the nucleus, 18% proteins are present in various cell organelles and 13% proteins act as integral part of the cell membrane.

Transcription factors (TFs) are the master regulators of gene expression patterns in eukaryotes, and are responsible for facilitation of growth and development in plants [37]. dno-miR414 identified in this study has been shown to target several transcription factors including those from MYB as well as TCP family of TFs. Members of MYB DNA-binding domain superfamily protein are involved in many important biochemical and physiological processes in plants [38]. Furthermore, previous studies have also reported that miR414 can target the MYB family transcription factors in Allium cepa, Solanum tuberosum and Brachypodium distachyon [39-41]. The plant-specific TCP (TEOSINTE BRANCHED 1, CYCLOIDEA, PCF 1 and 2) transcription factor family is involved in plant development throughout its vegetative phase, i.e. from seed germination until the formation of flowers and fruits [42]. Members of a few other families of transcription factors have also been found to be probable targets of dno-miR414, such as ERF, GATA and WRKY family of transcription factors. The ERF (Ethylene responsive) transcription factors are responsible for establishment of floral meristem and tissue repair processes [43]. GATA transcription factors (binding to GATA rich sequences) are the DNA motifs that have been mostly implicated in light-dependent gene regulation in plants [44],and the WRKY family of transcription factors has a significant role in regulation of abiotic stress responses in plants [45]. Our results also show that dno-miR414 and dno-miR528a may also target several genes which encode various F-box proteins. These proteins are characterized as components of the SCF ubiquitin-ligase complexes (Skp I, Cullin, and an F-box protein), in which they bind substrates for ubiquitin-mediated proteolysis [46]. Protein ubiquitination is considered as a critical post-translational modification process that is employed by eukaryotes in order to regulate various types of cellular processes [47]. Another important gene found to be targeted by dno-miR528 family is the co-chaperone that assists in protein folding mediated by HSP70 or HSP90 [48]. The KEGG pathway analysis also reveals involvement of miRNAs in regulation of genes associated with various significant metabolic pathways. Our findings have shown that ESTs can be a major source of functional information similar to previous reports of SSRs identified from ESTs [49].

## Conclusions:

We report the mining of miRNAs from EST data in Dendrobium nobile. We describe 14 potential miRNAs from 3 ESTs of D. nobile. They belong to 3 miRNA families (miR390, miR528 and miR414) linking to transcription factor regulation, signal transduction, DNA and protein binding, and various cellular processes covering 34 different metabolic networks in KEGG. These results help in the understanding of miRNA-mRNAs functional networks in Dendrobium nobile.

## Figures and Tables

**Table 1:** Identified putative miRNAs of D.nobile from ESTs

Accession no.	miRNA	Mature miRNA sequence	PL*	(C+G)%	MFE	AMFE	MFEI
HO190899.1	zma-miR528a-5p	TGGAAGGGGCATGCAGAGGAG	79	51.3	35.5	46.71	0.91
	zma-miR528b-5p	TGGAAGGGGCATGCAGAGGAG	79	51.3	35.5	46.71	0.91
HO191179.1	>cca-miR390	AAGCTCAGGAGGGATAGCG	107	43	42.55	39.76	0.92
	>lus-miR390a	AAGCTCAGGAGGGATAGCGCC	106	43.4	42.95	40.51	0.93
	>lus-miR390b	AAGCTCAGGAGGGATAGCGCC	106	43.4	42.95	40.51	0.93
	>csi-miR390b-5p	AGCTCAGGAGGGATAGCGCC	105	43.81	41.65	39.66	0.9
	>lus-miR390c	AAGCTCAGGAGGGATAGCGCC	106	43.4	42.95	40.51	0.93
	>ppt-miR390c-5p	AGCTCAGGAGGGATAGCGCC	105	43.81	41.65	39.66	0.9
	>lus-miR390d	AAGCTCAGGAGGGATAGCGCC	106	43.4	42.95	40.51	0.93
	>gma-miR390e	AGCTCAGGAGGGATAGCGCC	105	43.81	41.65	39.66	0.9
	>gma-miR390f	AAGCTCAGGAGGGATAGCGCC	106	43.4	42.95	40.51	0.93
	>gma-miR390g	AAGCTCAGGAGGGATAGCGCC	106	43.4	42.95	40.51	0.93
	>atr-miR390.1	TAAAGCTCAGGAGGGATAGCG	111	41.44	45.25	40.76	0.98
HO194934.1	>ath-miR414	GACGATGATGATGAAGATGA	169	47.93	47.8	28.28	0.59
Precursor Length

**Table 2:** Predicted target genes of D. nobile miRNAs.

miRNA_Acc.	Target_Acc.	Expect	Description	Inhibition
dno-miR390	AT3G17185.1	3	predicted protein	Cleavage
dno-miR390	AT5G03640.1	3	serine/threonine-protein kinase	Translation
dno-miR390.1	AT1G05500.1	3	synaptotagmin-5	Cleavage
dno-miR390.1	AT1G78950.1	3	beta-amyrinsynthase	Cleavage
dno-miR390.1	AT2G41600.5	3	Mitochondrial glycoprotein family	Cleavage
dno-miR390.1	AT4G12980.1	3	cytochrome b561 and DOMON domain-containing protein At4g12980	Cleavage
dno-miR390.1	AT5G11700.1	3	ephrin type-B receptor	Cleavage
dno-miR390.1	AT5G39862.1	3	putative non-LTR retroelement reverse transcriptase	Cleavage
dno-miR390b	AT5G48480.1	3	Lactoylglutathionelyase / glyoxalase I family protein	Cleavage
dno-miR390b-5p	AT3G52890.2	3	serine/threonine-protein kinase	Cleavage
dno-miR390c-5p	AT1G47890.1	3	receptor-like protein 12	Cleavage
dno-miR390e	AT5G05570.1	2.5	transducin family protein / WD-40 repeat family protein	Cleavage
dno-miR390e	AT5G05570.2	2.5	transducin family protein / WD-40 repeat family protein	Cleavage
dno-miR390e	AT3G11050.1	3	putative ferritin subunit precursor	Cleavage
dno-miR390f	AT4G32820.1	3	Tetratricopeptide repeat (TPR)-like superfamily protein	Cleavage
dno-miR390f	AT4G32820.2	3	Tetratricopeptide repeat (TPR)-like superfamily protein	Cleavage
dno-miR414	AT1G74890.1	0.5	two-component response regulator ARR15-like	Cleavage
dno-miR414	AT1G80960.2	1	F-box and Leucine Rich Repeat domains containing protein	Cleavage
dno-miR414	AT3G59220.1	1	PRN1_ARATHRecName: Full=Pirin-1; AltName: Full=AtPirin1	Cleavage
dno-miR414	AT5G20370.1	1	serine-rich protein-like protein	Cleavage
dno-miR414	AT5G23240.1	1	DNAJ heat shock N-terminal domain-containing protein	Cleavage
dno-miR414	AT5G61510.1	1	GroES-like zinc-binding alcohol dehydrogenase family protein	Cleavage
dno-miR414	AT1G05490.1	1.5	SNF2 domain-containing protein CLASSY 3-like	Cleavage
dno-miR414	AT1G45160.1	1.5	Protein kinasesuperfamily protein	Cleavage
dno-miR414	AT1G48970.1	1.5	translation initiation factor eIF-2B subunit delta	Cleavage
dno-miR414	AT1G53770.2	1.5	O-fucosyltransferase family protein	Cleavage
dno-miR414	AT1G78270.1	1.5	UDP-glycosyltransferase 85A4	Cleavage
dno-miR414	AT2G15345.1	1.5	Plant invertase/pectin methylesterase inhibitor superfamily protein	Cleavage
dno-miR414	AT2G17525.1	1.5	pentatricopeptide repeat-containing protein At2g17525, mitochondrial	Cleavage
dno-miR414	AT2G22000.1	1.5	elicitor peptide 6 precursor	Cleavage
dno-miR414	AT2G30790.1	1.5	oxygen-evolving enhancer protein 2-1, chloroplastic	Cleavage
dno-miR414	AT2G35960.1	1.5	NDR1/HIN1-like protein 12	Cleavage
dno-miR414	AT2G35970.1	1.5	NDR1/HIN1-like protein 12	Cleavage
dno-miR414	AT2G36460.2	1.5	Aldolasesuperfamily protein	Cleavage
dno-miR414	AT3G13730.1	1.5	3-epi-6-deoxocathasterone 23-monooxygenase	Cleavage
dno-miR414	AT3G17100.1	1.5	transcription factor bHLH147-like	Cleavage
dno-miR414	AT3G27640.1	1.5	denticleless protein homolog	Cleavage
dno-miR414	AT3G43590.1	1.5	protein AIR1	Cleavage
dno-miR414	AT4G16790.1	1.5	glycoprotein homolog	Cleavage
dno-miR414	AT1G22850.1	2	SNARE associated Golgi protein family	Cleavage
dno-miR414	AT1G26780.2	2	transcription factor MYB117	Cleavage
dno-miR414	AT1G75180.2	2	Erythronate-4-phosphate dehydrogenase family protein	Cleavage
dno-miR414	AT2G36320.1	2	zinc finger A20 and AN1 domain-containing stress-associated protein 6-like	Cleavage
dno-miR414	AT3G13930.1	2	dihydrolipoyllysine-residue acetyltransferase component 2 of pyruvatedehydrogenase complex	Cleavage
dno-miR414	AT3G21380.1	2	jacalin-related lectin 36	Cleavage
dno-miR414	AT4G32300.1	2	G-type lectin S-receptor-like serine/threonine-protein kinase SD2-5	Cleavage
dno-miR414	AT4G37630.1	2	cyclin d5	Cleavage
dno-miR414	AT4G39410.1	2	probable WRKY transcription factor 13	Cleavage
dno-miR414	AT5G11720.1	2	alpha-glucosidase	Cleavage
dno-miR414	AT1G05310.1	2.5	probable pectinesterase 8	Cleavage
dno-miR414	AT1G13430.1	2.5	P-loop containing nucleoside triphosphatehydrolasessuperfamily protein	Cleavage
dno-miR414	AT1G14920.1	2.5	DELLA protein GAI	Cleavage
dno-miR414	AT1G15710.1	2.5	Arogenatedehydrogenase 2, chloroplastic	Cleavage
dno-miR414	AT1G21326.1	2.5	Nuclear speckle RNA-binding protein B	Cleavage
dno-miR414	AT1G26390.1	2.5	berberine bridge enzyme-like 4	Cleavage
dno-miR414	AT1G28450.1	2.5	agamous-like MADS-box protein AGL29	Cleavage
dno-miR414	AT1G44830.1	2.5	ethylene-responsive transcription factor ERF014	Cleavage
dno-miR414	AT1G51640.1	2.5	exocyst complex component EXO70A1	Cleavage
dno-miR414	AT1G52160.1	2.5	tRNase Z TRZ3, mitochondrial	Cleavage
dno-miR414	AT1G54160.1	2.5	nuclear transcription factor Y subunit A-5	Cleavage
dno-miR414	AT1G60940.1	2.5	serine/threonine-protein kinase SRK2A	Cleavage
dno-miR414	AT1G66090.1	2.5	Disease resistance protein (TIR-NBS-LRR class) family	Cleavage
dno-miR414	AT1G68720.1	2.5	tRNA(adenine(34)) deaminase, chloroplastic	Cleavage
dno-miR414	AT1G69690.1	2.5	transcription factor TCP15-like	Cleavage
dno-miR414	AT1G71220.2	2.5	UDP-glucose:glycoproteinglucosyltransferase	Cleavage
dno-miR414	AT2G01530.1	2.5	MLP-like protein 328	Cleavage
dno-miR414	AT2G04620.1	2.5	zinc transporter-like protein	Cleavage
dno-miR414	AT2G06850.1	2.5	xyloglucanendotransglucosylase/hydrolase	Cleavage
dno-miR414	AT2G21530.1	2.5	SMAD/FHA domain-containing protein	Cleavage
dno-miR414	AT2G23530.1	2.5	cell division cycle-associated protein 7	Cleavage
dno-miR414	AT2G25110.1	2.5	stromal cell-derived factor 2-like protein	Cleavage
dno-miR414	AT2G28610.1	2.5	WUSCHEL-related homeobox 3	Cleavage
dno-miR414	AT2G32310.1	2.5	CCT motif family protein	Cleavage
dno-miR414	AT2G35110.2	2.5	protein NAP1 isoform X1	Cleavage
dno-miR414	AT2G37410.1	2.5	mitochondrial import inner membrane translocase subunit TIM17-2-like	Cleavage
dno-miR414	AT2G43970.1	2.5	la-related protein 6B	Cleavage
dno-miR414	AT2G43970.2	2.5	la-related protein 6B	Cleavage
dno-miR414	AT2G45880.1	2.5	beta-amylase 7	Cleavage
dno-miR414	AT2G47350.2	2.5	HIT zinc finger and PAPA-1-like domain-containing protein	Cleavage
dno-miR414	AT2G47830.1	2.5	metal tolerance protein C1	Cleavage
dno-miR414	AT3G01770.1	2.5	transcription factor GTE9 isoform X1	Cleavage
dno-miR414	AT3G01830.1	2.5	probable calcium-binding protein CML40	Cleavage
dno-miR414	AT3G02150.1	2.5	transcription factor TCP13	Cleavage
dno-miR414	AT3G02150.2	2.5	transcription factor TCP13	Cleavage
dno-miR414	AT3G22770.1	2.5	putative F-box protein At3g23420	Cleavage
dno-miR414	AT3G23270.1	2.5	Regulator of chromosome condensation (RCC1) family with FYVE zinc finger domain-containing protein	Cleavage
dno-miR414	AT3G24650.1	2.5	B3 domain-containing transcription factor ABI3	Cleavage
dno-miR414	AT3G45190.1	2.5	serine/threonine-protein phosphatase 6 regulatory subunit 3-like isoform X1	Cleavage
dno-miR414	AT3G49350.1	2.5	GTPase-activating protein gyp7	Cleavage
dno-miR414	AT3G54920.1	2.5	probable pectatelyase 13	Cleavage
dno-miR414	AT3G60790.1	2.5	F-box protein At3g60790-like	Cleavage
dno-miR414	AT4G03030.1	2.5	F-box/kelch-repeat protein OR23	Cleavage
dno-miR414	AT4G11600.1	2.5	probable phospholipidhydroperoxide glutathione peroxidase 6, mitochondrial	Cleavage
dno-miR414	AT4G18390.1	2.5	transcription factor TCP2	Cleavage
dno-miR414	AT4G18780.1	2.5	cellulose synthase A catalytic subunit 8 [UDP-forming]	Cleavage
dno-miR414	AT4G19830.1	2.5	peptidyl-prolylcis-trans isomerase FKBP17-1, chloroplastic	Cleavage
dno-miR414	AT4G23680.1	2.5	MLP-like protein 328	Cleavage
dno-miR414	AT4G24340.1	2.5	Phosphorylasesuperfamily protein	Cleavage
dno-miR414	AT4G27320.1	2.5	universal stress protein PHOS34	Cleavage
dno-miR414	AT4G28620.1	2.5	ABC transporter B family member 23, mitochondrial	Cleavage
dno-miR414	AT4G29180.1	2.5	root hair specific 16	Cleavage
dno-miR414	AT4G29180.2	2.5	root hair specific 16	Cleavage
dno-miR414	AT4G30600.1	2.5	signal recognition particle receptor subunit alpha-like	Cleavage
dno-miR414	AT4G34390.1	2.5	extra-large GTP-binding protein 2	Cleavage
dno-miR414	AT4G35900.1	2.5	bZIP transcription factor	Cleavage
dno-miR414	AT5G03340.1	2.5	cell division control protein 48 homolog E	Cleavage
dno-miR414	AT5G03545.1	2.5	expressed in response to phosphate starvation protein	Cleavage
dno-miR414	AT5G13640.1	2.5	phospholipid:diacylglycerolacyltransferase 1	Cleavage
dno-miR414	AT5G16830.1	2.5	syntaxin-21	Cleavage
dno-miR414	AT5G40630.1	2.5	BAG family molecular chaperone regulator 2	Cleavage
dno-miR414	AT5G41410.1	2.5	homeobox protein BEL1 homolog	Cleavage
dno-miR414	AT5G42780.1	2.5	zinc-finger homeodomain protein 13	Cleavage
dno-miR414	AT5G47220.1	2.5	ethylene responsive element binding factor 2 (ATERF2)	Cleavage
dno-miR414	AT5G48380.1	2.5	probably inactive leucine-rich repeat receptor-like protein kinase At5g48380	Cleavage
dno-miR414	AT5G49740.1	2.5	ferric reduction oxidase 7, chloroplastic	Cleavage
dno-miR414	AT5G53730.1	2.5	NDR1/HIN1-like protein 12	Cleavage
dno-miR414	AT5G56040.1	2.5	probable LRR receptor-like serine/threonine-protein kinase At4g26540	Cleavage
dno-miR414	AT5G56860.1	2.5	GATA transcription factor 21-like	Cleavage
dno-miR414	AT5G59030.1	2.5	copper transporter 1	Cleavage
dno-miR414	AT1G12760.1	3	Zinc finger, C3HC4 type (RING finger) family protein	Cleavage
dno-miR414	AT1G19770.1	3	probable purinepermease 14	Cleavage
dno-miR414	AT1G68550.2	3	ethylene-responsive transcription factor ERF118-like	Translation
dno-miR414	AT1G68552.1	3	ethylene-responsive transcription factor ERF118-like	Translation
dno-miR414	AT1G69935.1	3	protein SHORT HYPOCOTYL IN WHITE LIGHT 1	Cleavage
dno-miR414	AT2G23810.1	3	tetraspanin-8	Cleavage
dno-miR414	AT2G42710.1	3	Ribosomal protein L1p/L10e family	Cleavage
dno-miR414	AT4G31180.1	3	aspartate--tRNAligase 2, cytoplasmic	Cleavage
dno-miR414	AT5G50210.1	3	quinolinatesynthase, chloroplastic	Cleavage
dno-miR414	AT5G67520.1	3	adenosine-5'-phosphosulfate (APS) kinase 4	Cleavage
dno-miR528a-5p	AT4G32770.1	2.5	tocopherolcyclase, chloroplastic	Cleavage
dno-miR528a-5p	AT1G80370.1	3	cyclin-A2-4-like	Cleavage
dno-miR528a-5p	AT2G40920.1	3	F-box/LRR-repeat protein	Cleavage
dno-miR528a-5p	AT5G62380.1	3	NAC domain-containing protein 101-like	Cleavage
dno-miR528b-5p	AT5G17710.2	3	Co-chaperone GrpE family protein	Cleavage
